# LightDPH: Lightweight Dual-Projection-Head Hierarchical Contrastive Learning for Skin Lesion Classification

**DOI:** 10.1007/s41666-024-00174-5

**Published:** 2024-10-01

**Authors:** Benny Wei-Yun Hsu, Vincent S. Tseng

**Affiliations:** 1 Institute of Computer Science and Engineering, National Yang Ming Chiao Tung University, No. 1001, Daxue Rd., Hsinchu City, 300093 Taiwan Republic of China; 2 Department of Computer Science, National Yang Ming Chiao Tung University, No. 1001, Daxue Rd., Hsinchu City, 300093 Taiwan Republic of China

**Keywords:** Lightweight model, Contrastive learning, Skin lesion classification, Hierarchical knowledge, Deep learning

## Abstract

Effective skin cancer detection is crucial for early intervention and improved treatment outcomes. Previous studies have primarily focused on enhancing the performance of skin lesion classification models. However, there is a growing need to consider the practical requirements of real-world scenarios, such as portable applications that require lightweight models embedded in devices. Therefore, this study aims to propose a novel method that can address the major-type misclassification problem with a lightweight model. This study proposes an innovative Lightweight Dual Projection-Head Hierarchical contrastive learning (LightDPH) method. We introduce a dual projection-head mechanism to a contrastive learning framework. This mechanism is utilized to train a model with our proposed multi-level contrastive loss (MultiCon Loss), which can effectively learn hierarchical information from samples. Meanwhile, we present a distance-based weight (DBW) function to adjust losses based on hierarchical levels. This unique combination of MultiCon Loss and DBW function in LightDPH tackles the problem of major-type misclassification with lightweight models and enhances the model’s sensitivity in skin lesion classification. The experimental results demonstrate that LightDPH significantly reduces the number of parameters by 52.6% and computational complexity by 29.9% in GFLOPs while maintaining high classification performance comparable to state-of-the-art methods. This study also presented a novel evaluation metric, model efficiency score (MES), to evaluate the cost-effectiveness of models with scaling and classification performance. The proposed LightDPH effectively mitigates major-type misclassification and works in a resource-efficient manner, making it highly suitable for clinical applications in resource-constrained environments. To the best of our knowledge, this is the first work that develops an effective lightweight hierarchical classification model for skin lesion detection.

## Introduction

Skin cancer is a malignant disease that can lead to severe consequences, including death. Fortunately, the majority of skin cancer cases, such as basal cell carcinoma (BCC) and squamous cell carcinoma (SCC), can be cured if detected and diagnosed at an early stage [1]. By closely monitoring any suspicious skin developments and promptly seeking professional medical attention when necessary, people can significantly increase their chances of successful treatment and recovery. To this end, it is essential to possess a vigilant approach to detecting skin cancer. Deep learning has been widely applied to medical fields [2], aiming to improve clinical workflows.

In the meantime, combining deep learning technology with skin lesion analysis is a highly sought-after research topic in medical applications [3–9].

While previous studies of skin lesion classification primarily focused on performance improvement, there is a growing need to take into account the demands of real-world scenarios. For instance, the recent development of portable devices, such as digital dermatoscopes, for the rapid imaging of skin spots has emphasized the need for accurate and practical classification systems that can be effectively deployed in daily use. Despite significant advances in developing high-accuracy classification models for skin lesion analysis, current methods typically rely on ensemble techniques that combine multiple models [10–12] leading to large storage requirements. This limitation has revealed a necessity for alternative methods that can achieve similar levels of accuracy with reduced storage demands.

In addition to ensemble methods, recent studies have highlighted the potential of hierarchical information to enhance accuracy in various tasks, including classification [13, 14], image retrieval [15], and video understanding [16]. In skin lesion classification, the hierarchical approach has shown promising results. In the research of hierarchy-based methods, a major-type correctness problem in multi-class classification has been raised [10], which is an essential issue for clinical applications. The primary objective of skin lesion classification is to identify the lesion classes accurately. However, as the number of classes increases, it becomes more challenging for machine learning models to correctly classify skin lesions. Therefore, it is crucial to ensure that misclassified samples are categorized into their major types as accurately as possible to prevent missed diagnoses. For example, the major types are benign and malignant/premalignant lesions. Given a skin lesion image of melanoma, if a multi-class classifier misclassifies the image, we hope the classifier classifies it to a subclass of the same major type (i.e., the malignant class). Though the hierarchy-aware contrastive learning with late fusion method (HAC-LF) [10] has addressed the problem, it still counts on ensemble techniques for better classification performance.

To address the aforementioned challenges, this study presents a novel lightweight skin lesion classification model suitable for further clinical applications, which maintains high accuracy while minimizing storage requirements. Additionally, we address the issue of major-type misclassification in skin lesion classification by introducing hierarchical knowledge to the proposed method. In summary, this research offers the following significant contributions.

- This study proposes a novel method, Lightweight Dual Projection-Head Hierarchical contrastive learning (LightDPH), to construct lightweight inference models suitable for practical use.
- This study presents a new loss function, multi-level contrastive loss (MultiCon Loss), incorporating hierarchical knowledge to mitigate major-type misclassification problems.
- This study introduces the model efficiency score (MES), a new metric suitable for assessing the cost-effectiveness of increasing the model scale for improved classification performance.
- Our experimental results demonstrate the superiority of the proposed method over state-of-the-art hierarchical contrastive learning methods in skin lesion classification, considering the model scale and classification performance.

This paper is organized as follows. Section 2 provides details of our proposed methods, and Sect. 3 demonstrates the results with comprehensive experiments and analyses. Finally, in Sect. 4 and Sect. 5, we present the discussion and conclusion, respectively.

## Proposed Methods

**Fig. 1:**
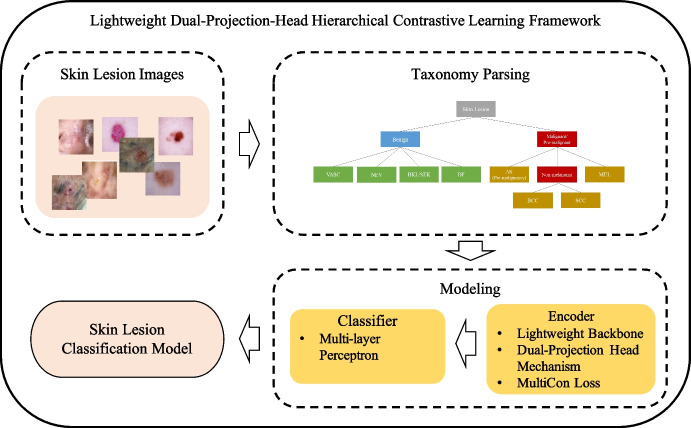
Framework of the lightweight skin lesion classification model

This study aims to develop a skin lesion classification model that balances classification performance (e.g., accuracy and sensitivity) and model scale for portable use. Thus, we have introduced the Lightweight Dual Projection-Head Hierarchical (LightDPH) contrastive learning method. This method incorporates a novel learning architecture and loss functions designed to exploit hierarchical knowledge for optimizing the model scale and enhancing skin lesion classification performance. Central to the LightDPH framework (Fig. 1) is a hierarchy parsing stage that processes skin lesion images using a pre-defined hierarchy similar to that reported in previous studies [17]. This hierarchical structure aids in systematically decoding the complex relationships among different types of skin lesions, such as malignant tumors. By integrating hierarchical category information, our model learns generic features and captures shared semantic information across different malignant tumor types. This approach significantly lowers the risk of misclassifying malignant conditions as benign, thus markedly improving the detection of anomalies. In the modeling stage, we train an encoder and a classifier separately; the encoder utilizes contrastive learning with a lightweight backbone, and the classifier consists of a multi-layer perceptron with two hidden layers. Once completed, the modeling process culminates in a robust skin lesion classification model ready for evaluation and practical deployment.

### Architecture of LightDPH

**Fig. 2:**
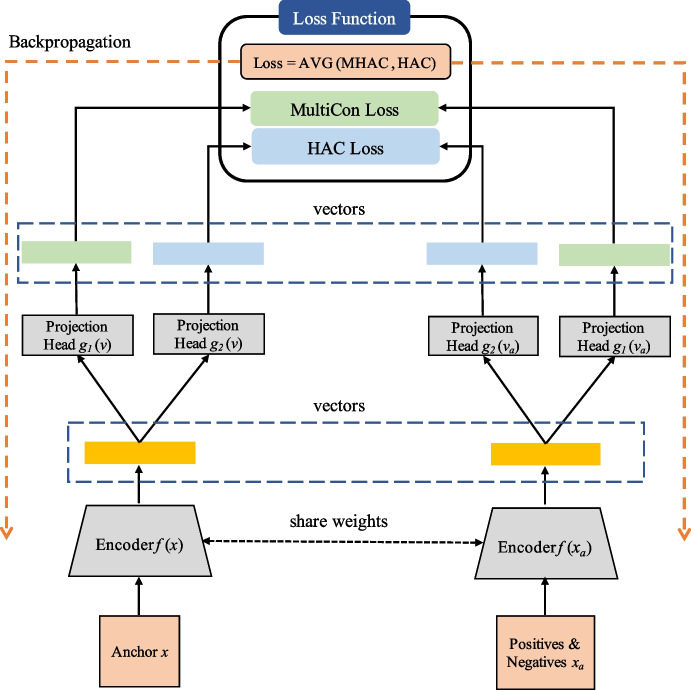
Architecture of lightweight dual-projection-head hierarchical contrastive learning

The architecture of LightDPH is depicted in Fig. 2. LightDPH addresses three crucial problems of existing works. Firstly, to enhance the classification performance without ensemble models, we introduce a novel architecture with dual projection heads for different learning purposes in the training phase, replacing the generic architecture used in previous works [10, 14, 18, 19]. The projection head receives the features from the backbone network and projects them to lower-dimensional features for further loss calculation. While ensemble methods aim to aggregate multiple models to increase performance, unsystematic combinations of models are inefficient. Although the previous work, HAC-LF [10], targeted hierarchical information for model fusion and achieved promising results for skin lesion classification, it still consists of two encoders. Therefore, the proposed dual projection heads offer a simple but effective refinement to the HAC-LF architecture. As depicted in Fig. 2, the input data $\{x_k\}_{k=1}^N$, where *N* is the batch size, is processed by an encoder $f(\cdot )$ that maps $x_k$ to a vector $v_k \in \mathbb {R^{D_V}}$, where $D_V = 1000$. During training, two projection heads, $g_1(\cdot )$ and $g_2(\cdot )$, are used to project $v_k$ to a vector $z_k \in \mathbb {R^{D_Z}}$, where $D_Z = 128$. The first projection head, $g_1(\cdot )$, generates a feature vector for MultiCon loss calculations, while the second projection head, $g_2(\cdot )$, generates a vector for HAC loss calculation that focuses on the relationship among leaf-node classes. The final loss is the average of the two losses. The projection heads are MLPs with one hidden layer and are removed during downstream task training and validation/testing phases. Thus, only $f(x_k)$ is used for further classifier training after representation learning. We use an MLP with two hidden layers as our classifier for the downstream tasks. The input vector of the classifier is the output of the encoder $f(\cdot )$ with frozen weights.

Secondly, LightDPH addresses the issue of model scale by utilizing a lightweight backbone network to generate representations of input images. CNN has proven effective in various fields. While performance and the number of parameters are not always positively correlated in some applications, studies on general image classification benchmarks using CNN-based methods have demonstrated a positive correlation between them [20–22]. To this end, we adopt the EfficientNet-B0 to B4 models as the lightweight backbone networks (i.e., encoders) in LightDPH. More details are provided in the Sect. 2.2.

Finally, LightDPH tackles the problem of major-type misclassification by leveraging hierarchical knowledge. We define the hierarchy of skin lesions in advance to take advantage of this knowledge. The hierarchy parsing stage processes skin lesion images and extracts hierarchical features for further modeling. The loss functions for hierarchical knowledge extraction help to balance the model scale and skin lesion classification performance.

Overall, LightDPH offers a lightweight, hierarchy-aware, and effective skin lesion classification model for realistic applications. It achieves state-of-the-art performance on benchmark datasets and provides a promising solution for portable and accurate skin lesion classification.

### Backbone Networks

In this study, our aim is to enable models to learn crucial information that enhances their performance with fewer parameters. To achieve this goal, we focus on using CNN-based methods to construct the backbone networks, rather than transformer-based and hybrid methods (i.e., combinations of transformers and CNNs), which have been shown to produce powerful classification performance on image classification benchmarks but consist of billions of parameters [23–26]. Considering the accuracy and number of parameters of existing CNN-based methods, we employ the EfficientNetB0-B4 architecture [20] as our backbone networks. The EfficientNet family has demonstrated remarkable results on the image classification benchmark [27]. Compared to the top methods on the leaderboard, transformer-based and hybrid methods typically consist of 1.8 to 3.9 billion parameters, while the model proposed in [28], based on EfficientNet backbone, achieved 90.2% accuracy with only 480 million parameters. Therefore, we adopt the EfficientNet architecture as our backbone network for this study.

### Loss Function

#### Hierarchy-Aware Contrastive Loss

**Fig. 3:**
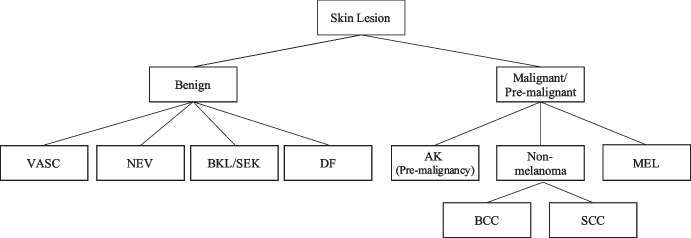
Hierarchical classification structures for skin lesions

Contrastive learning has gained prominence in various applications since it demonstrated the flexibility of different downstream tasks with effective representation learning strategies [29–31]. In order to reduce the model scale and enhance classification performance through learning hierarchical knowledge, we address the problem by improving the hierarchy-aware contrastive loss function (HAC Loss) [10], which is defined as follows:

1 $$\begin{aligned} Loss_{HAC} = -\sum _{i=1}^{2N} \frac{\sum _{p\in P(i)} hrchy(i,p) \log \frac{\exp (z_i \cdot z_p/\tau )}{{\sum _{a \in A(i)}\exp (z_i \cdot z_a/\tau ))}}}{{\sum _{p\in P(i)} hrchy(i,p)}} \end{aligned}$$

where *N* represents the batch size, and $i \in {1,...,2N}$ is a set of indices for input images and their augmented ones. $A(i) = {1,...,2N} \setminus {i}$ denotes all indices except the one of anchor images $x_i$. $P(i)={p \in A(i):major(\bar{y_i}) = major(\bar{y_p}) }$ represents a set of positive images, where the function $major(\bar{y})$ returns the superclass of the predicted class vector $\bar{y}$. The output vector from an encoder is represented by $z_k$, and $\tau \in \mathbb {R^+}$ is a scalar temperature parameter. $hrchy(\cdot )$ is an essential weight function that introduces hierarchical information to the learning procedure. The original $hrchy(\cdot )$ is defined as follows:

2 $$\begin{aligned} hrchy_{origin}(i, p) = {\left\{ \begin{array}{ll} 1, if \ \bar{y_i} = \bar{y_p} \\ h \in \mathbb {R^+}, if \ \bar{y_i} \ne \bar{y_p}\ \\ \end{array}\right. } \end{aligned}$$

where *h* is a hyperparameter. However, the current *hrchy*(*i*, *p*) only takes into account the relationship between major-type classes and leaf-node classes, thereby limiting its ability to extract detailed information from intermediate nodes in a multi-level hierarchy. An example of such a hierarchy can be seen in Fig. 3, which depicts the skin lesion hierarchy used in our study. The hierarchy shows that the weight assigned to melanoma and non-melanoma cancer (e.g., SCC) is the same and determined by *h*. To overcome this limitation and incorporate more hierarchical knowledge to handle multi-level structures, we propose a distance-based weight function to adjust the importance of contrastive pairs.

#### Multi-level Contrastive Loss

**Fig. 4:**
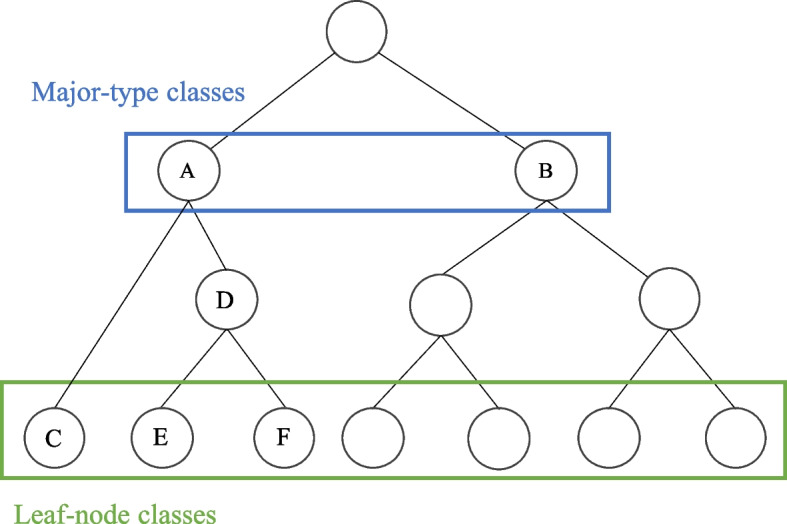
Example of hierarchical classification used for distance-based weight calculation

We introduce multi-level contrastive loss (MultiCon Loss) by incorporating our proposed distance-based weight function into HAC Loss. The distance-based weight function replaces the original $hrchy(\cdot )$, enabling the learning of more information between two samples belonging to the same major type. An example of a hierarchical classification structure is presented in Fig. 4, where *A* and *B* are major-type classes, and *C*, *E*, and *F* are leaf-node classes under the major-type class *A*. In cases where a sample belongs to class *E*, the distance of (*E*, *C*) differs from the distance of (*E*, *F*), even though they belong to the same major type. To address this issue, we propose a distance-based weight function $DBW(\cdot )$ to modify $hrchy(\cdot )$ defined as follows:

3 $$\begin{aligned} DBW(N_i, N_j) = \frac{k \times h}{d(N_i)+d(N_j)-2d(LCA(N_i, N_j))} \end{aligned}$$

where $N_i$ and $N_j$ are leaf-node classes that belong to the same major type, $N_i \ne N_j$, $k \in \mathbb {N^+}$, $h \in \mathbb {R^+}$, $d(\cdot )$ returns the number of edges between the root and a node (i.e., the distance from a node to the root in a graph), and $LCA(\cdot )$ calculates lowest common ancestor of two nodes. Thus, the new $hrchy(\cdot )$ is defined as follows:

4 $$\begin{aligned} hrchy_{new}(i, p) = {\left\{ \begin{array}{ll} 1, if \ \bar{y_i} = \bar{y_p} \\ DBW(N_i, N_p), if \ \bar{y_i} \ne \bar{y_p}\ \\ \end{array}\right. } \end{aligned}$$

As discussed in Sect. 2.1, the cumulative loss is computed by averaging the MultiCon Loss and HAC Loss. For HAC Loss ($h = 1$), prioritizing the contrastive correlation between all leaf-node classes, irrespective of their major-type classes.

## Experimental Evaluation

### Datasets

In order to evaluate skin lesion classification performance, this study uses two datasets. The first, ISIC 2019, is a comprehensive collection of dermoscopic images of skin lesions that includes the HAM10000 [32], BCN20000 [33], and MSK datasets [34]. The ISIC 2019 dataset features 25,331 expertly annotated images from 12,795 patients obtained from various sources such as hospitals, clinics, and private practices. The skin lesions in the ISIC 2019 dataset encompass actinic keratosis (AK), basal cell carcinoma (BCC), melanoma (MEL), squamous cell carcinoma (SCC), benign keratosis (BKL), dermatofibroma (DF), melanocytic nevus (NV), and vascular lesions (VASC). This dataset has become a standard benchmark for skin lesion classification and segmentation tasks and has catalyzed the development of numerous state-of-the-art techniques in dermatology. The second dataset used in this study is PAD-UFES-20 [35], which contains skin lesion images collected from smartphones. PAD-UFES-20 includes 2298 samples of six different types of skin lesions from 1373 patients. The skin lesions in the PAD-UFES-20 dataset are BCC, SCC, AK, MEL, NV, and seborrheic keratosis (SEK). In this study, we take malignant and premalignant lesions as the same major type since the premalignant lesion should be cared for and follow-up. Table 1 showcases the data distribution of the two used datasets.

**Table 1:** Data distribution of PAD-UFES-20 and ISIC 2019

Major type	Classes	PAD-UFES-20	ISIC 2019
Malignant and premalignant	AK	730	867
	BCC	845	3323
	MEL	52	4522
	SCC	192	628
Benign	BKL	–	2624
	DF	–	239
	NV	244	12,875
	SEK	235	–
	VASC	–	253

### Experimental Setting

This study employs a stratified fivefold cross-validation approach to conduct a series of experiments to evaluate the performance of the proposed method and compare it with the state-of-the-art methods. The evaluation metrics used in this study are accuracy, sensitivity, and specificity, defined as follows:

5 $$\begin{aligned} Accuracy = \frac{TP+TN}{TP+FP+TN+FN} \end{aligned}$$

6 $$\begin{aligned} Sensitivity = \frac{TP}{TP + FN} \end{aligned}$$

7 $$\begin{aligned} Specificity = \frac{TN}{TN + FP} \end{aligned}$$

where TP, TN, FP, and FN denote true positive, true negative, false positive, and false negative, respectively. In multi-class classification, we calculate the sensitivity for each class and average them, which is equivalent to balanced accuracy (BACC). BACC is defined as an average of sensitivity and specificity in binary classification, considering data imbalance problems. Therefore, to clarify the evaluation purpose, we use “sensitivity” in the performance comparison; “BACC” for the result analysis. To analyze the major-type misclassification problem, we also assess performance by utilizing major-type accuracy (M-Acc), major-type sensitivity (M-Sens), and major-type specificity (M-Spec). For instance, if a BCC (malignant lesion) sample was classified as SCC (malignant lesion), the result should be considered true positive.

In this study, we compute the number of parameters (Params) and giga floating-point operations (GFLOPs) to assess the inference models’ practical utility. We anticipate the backbone model scale to be small for applications. Thereby, we proposed a model efficiency score (MES) to measure the contribution of the increased parameters and floating-point operations for classification performance. Given a performance value $p \in [0,1]$, $P_B$ and $P_N$ are baseline performance $p_b \times \gamma$ and new model performance $p_n \times \gamma$, respectively. $\gamma$ is scaled according to the evaluation metric types. We set $\gamma$’s default to 100 to align with our main evaluation metrics, which range from 0 to 1, ensuring comparability. We define MES as below:

8 $$\begin{aligned} MES = \max (0, \frac{P_N-P_B}{MSV_N-MSV_B}) \end{aligned}$$

where $MSV_B$ and $MSV_N$ are model-scale-based variables (i.e., the number of parameters and floating-point operations in this work) of the baseline and new models. The model scale of baseline models should be smaller than new models while using MES. As $\frac{P_N-P_B}{MSV_N - MSV_B} < 0$, it reveals that new models have worse performance than baseline models. Hence, we adopted $\max (0,\cdot )$ to manifest that 0 is no contribution to performance improvement.

This study evaluates accuracy-related and model scales, such as parameter count and computational complexity. Thus, we employ relative changes (%), systematically depicting changes in both accuracy-related metrics and model size in percentage terms. This approach highlights the correlation between accuracy enhancements and reductions in model size and underscores the consistency of our evaluation criteria. The formula for calculating relative change is shown below:

9 $$\begin{aligned} \text {Relative Change (\%)} = \left( \frac{\text {New Value} - \text {Baseline Value}}{\text {Baseline Value}} \right) \times 100 \end{aligned}$$

We implemented our proposed method and compared methods using PyTorch 1.4.1. for training and testing on a server equipped with a Xeon Gold CPU, 128GB memory, and an Nvidia V100 GPU card. This server runs on an Ubuntu 18.04 system.

### Backbone Networks and Baseline Performance

In this section, we evaluate the classification performance and model scale of our proposed method using EfficientNet-B0 to B4 as baselines and backbone networks. The PAD-UFES-20 and ISIC 2019 datasets are utilized to compare the performance, as presented in Table 2. EfficientNets have been extensively evaluated on large-scale natural image datasets, such as ImageNet [36], where it has been observed that higher parameter counts lead to improved classification accuracy. However, when evaluating EfficientNet-B0 to B4 on skin lesion datasets of varying scales, it becomes evident that the relationship between network parameters and performance is only sometimes proportional in specific domains.

According to the results of the baseline model performance, it can be observed that among the EfficientNet models evaluated, B4 exhibits the highest number of parameters and floating-point operations. In spite of its larger model scale, B4 demonstrates relatively lower classification performance compared to other EfficientNet variants. The evaluation of ISIC 2019 and PAD-UFES-20 reveals similar results that B4 does not significantly outperform other networks.

**Table 2:** Performance comparison with the baseline models on PAD-UFES-20 and ISIC 2019

Dataset	Method	M-Acc	M-Sens	M-Spec	Acc	Sens	Spec	Params	GFLOPs
PAD-UFES-20	EfficientNet-B0	0.915	0.943	0.810	0.686	0.645	0.931	4.1M	0.42
	EfficientNet-B1	0.926	0.954	0.818	0.717	0.664	0.935	6.6M	0.62
	EfficientNet-B2	0.917	0.952	0.787	0.702	0.659	0.934	7.8M	0.71
	EfficientNet-B3	0.916	0.949	0.789	0.691	0.633	0.932	10.8M	1.03
	EfficientNet-B4	0.902	0.913	0.860	0.693	0.662	0.934	17.7M	1.59
ISIC 2019	EfficientNet-B0	0.852	0.791	0.887	0.765	0.636	0.959	4.1M	0.42
	EfficientNet-B1	0.849	0.819	0.867	0.758	0.661	0.960	6.6M	0.62
	EfficientNet-B2	0.856	0.804	0.886	0.776	0.666	0.962	7.8M	0.71
	EfficientNet-B3	0.848	0.736	0.913	0.747	0.619	0.955	10.8M	1.03
	EfficientNet-B4	0.858	0.794	0.895	0.780	0.661	0.957	17.7M	1.59

These intriguing findings underline that a larger number of parameters and floating-point operations do not necessarily guarantee improved classification performance, emphasizing the intricate relationship between model complexity and effectiveness. Hence, our objective is to optimize the trade-off between classification performance and inference model scale, ensuring practical usability and efficiency in real-world applications.

**Table 3:** Performance comparison of LightDPH and the state-of-the-art methods on PAD-UFES-20. Values in bold indicate the highest performance

Model	M-Acc	M-Sens	M-Spec	Acc	Sens	Spec	Params	GFLOPs
HAC-LF (B0)	0.939	0.969	0.827	0.726	0.672	0.938	12.7M	1.85
HAC-LF (B1)	0.939	0.961	**0**.**858**	0.737	0.663	0.941	17.7M	2.25
HAC-LF (B2)	0.943	0.973	0.829	0.735	0.663	0.940	20.4M	2.43
HAC-LF (B3)	0.941	0.963	**0**.**858**	**0.743**	0.666	0.942	26.7M	3.07
HAC-LF (B4)	0.936	0.960	0.841	0.732	0.659	0.940	42.0M	4.20
HMCE (B0)	0.939	0.964	0.845	0.714	0.653	0.935	6.3M	1.43
HMCE (B1)	0.941	0.967	0.843	0.729	0.646	0.934	8.9M	1.63
HMCE (B2)	0.945	0.970	0.848	0.715	0.648	0.935	10.2M	1.72
HMCE (B3)	0.940	0.969	0.833	0.713	0.657	0.936	13.3M	2.04
HMCE (B4)	0.939	0.967	0.833	0.711	0.644	0.935	20.5M	2.60
LightDPH (B0)	0.941	0.971	0.827	0.721	**0**.**692**	0.938	6.3M	1.43
LightDPH (B1)	0.940	0.966	0.841	0.726	0.683	0.939	8.9M	1.63
LightDPH (B2)	**0**.**949**	**0**.**974**	0.854	0.734	0.691	**0**.**942**	10.2M	1.72
LightDPH (B3)	0.936	0.964	0.827	0.724	0.683	0.939	13.3M	2.04
LightDPH (B4)	0.941	0.968	0.837	0.725	0.671	0.939	20.5M	2.60

The use of backbone models is provided in parentheses following the method name

### Performance Comparison with the State-of-the-Art Methods

The results presented in Table 3 show the comparison of our proposed LightDPH and the state-of-the-art methods using the PAD-UFES-20 dataset. The state-of-the-art methods include Hierarchy-Aware Contrastive Learning with Late Fusion (HAC-LF) [10] and Hierarchical Multi-label Constraint Enforcing Contrastive Loss (HMCE) [14]. HMCE shares a similar idea with our study, focusing on multi-label datasets. However, their methods were evaluated on the data where the number of ancestors for each leaf node needed to be the same, and it limited the flexibility of the hierarchical structure. All the baseline models were used as the backbone networks for each method. Table 3 indicates that all hierarchy-knowledge-based methods effectively address the major-type misclassification problems with better M-Spec while keeping or exceeding the M-Acc and M-Sens of the baseline models. In the multi-class classification results of Table 3, LightDPH (B0) can outperform the other methods on sensitivity (i.e., balanced accuracy). HAC-LF (B0) achieves its best sensitivity of 0.672 with 12.7M parameters and 1.85 GFLOPs, while LightDPH achieves a sensitivity of 0.692 with 6.3M parameters and 1.43 GFLOPs. Compared to HAC-LF (B0), LightDPH (B0) has relatively reduced 50.4% parameters and 22.7% GLOPs with a relative improvement of 3% sensitivity. Although HMCE (B3) achieves the best sensitivity in HMCE models, we can take HMCE (B0) as the best HMCE model rather than HMCE (B3) with the consideration of the balance between the model scale and classification performance. Thus, compared to HMCE (B0), LightDPH has relatively increased by 6% of sensitivity with the same model scale. If we compared LightDPH (B0) to the HMCE (B3), LightDPH (B0) has relatively reduced 52.6% parameters and 29.9% GLOPs with a relative improvement of 5% sensitivity.

In addition to the PAD-UFES-20 dataset, our study includes the evaluation of a large-scale skin lesion dataset, ISIC 2019, to assess the performance of the models. The experimental results on ISIC 2019 are presented in Table 4. The findings from the major-type classification reveal that hierarchical knowledge remains effective in addressing major-type misclassification, surpassing the performance of the baseline models. In the context of multi-class classification, complex models demonstrate their capabilities on large-scale datasets. However, our proposed LightDPH achieves comparable performance with significantly fewer model scales. For instance, when comparing LightDPH (B3) with the same backbone as HAC-LF (B3), the former achieves a sensitivity of 0.690 with 13.3M parameters and 2.04 GFLOPs, while the latter achieves a sensitivity of 0.709 with 26.7M parameters and 3.07 GLOPs. The results demonstrate LightDPH’s ability to save more than 50% of parameters and 33% of GLOPs while achieving similar performance.

**Table 4:** Performance comparison of LightDPH and the state-of-the-art methods on ISIC 2019. Values in bold indicate the highest performance

Method	M-Acc	M-Sens	M-Spec	Acc	Sens	Spec	Params	GFLOPs
HAC-LF (B0)	0.892	0.855	0.913	0.820	0.679	0.968	12.7M	1.85
HAC-LF (B1)	0.897	0.861	**0**.**918**	0.828	0.689	0.969	17.7M	2.25
HAC-LF (B2)	**0**.**898**	**0**.**871**	0.915	0.827	0.696	**0**.**970**	20.4M	2.43
HAC-LF (B3)	0.897	0.867	0.914	**0**.**829**	**0**.**709**	**0**.**970**	26.7M	3.07
HAC-LF (B4)	0.889	0.854	0.909	0.815	0.688	0.968	42.0M	4.20
HMCE (B0)	0.885	0.851	0.904	0.797	0.642	0.964	6.3M	1.43
HMCE (B1)	0.890	0.864	0.905	0.803	0.657	0.966	8.9M	1.63
HMCE (B2)	0.894	0.857	0.916	0.806	0.660	0.966	10.2M	1.72
HMCE (B3)	0.894	0.864	0.912	0.809	0.650	0.966	13.3M	2.04
HMCE (B4)	0.894	0.860	0.914	0.811	0.661	0.967	20.5M	2.60
LightDPH (B0)	0.886	0.849	0.908	0.811	0.675	0.967	6.3M	1.43
LightDPH (B1)	0.887	0.864	0.901	0.816	0.680	0.968	8.9M	1.63
LightDPH (B2)	0.893	0.859	0.913	0.814	0.684	0.968	10.2M	1.72
LightDPH (B3)	0.892	0.859	0.911	0.820	0.690	0.969	13.3M	2.04
LightDPH (B4)	0.890	0.857	0.910	0.815	0.692	0.968	20.5M	2.60

The use of backbone models is provided in parentheses following the method name

Based on these comparison results, it is important to consider the trade-off between model scale and classification performance in clinical practice. Accordingly, we conducted further analysis to assess the efficiency by evaluating the contribution of the number of parameters and GFLOPs to BACC. This analysis provides valuable insights into the practical utility of the models.

### Influence of Evaluation Metrics on Imbalanced Data

**Fig. 5:**
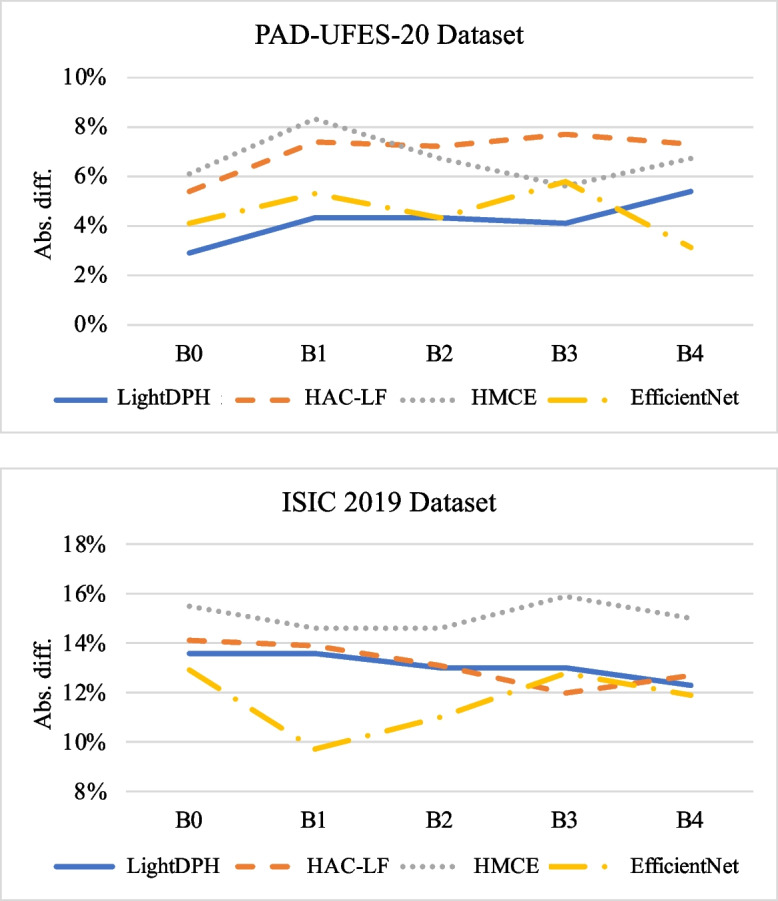
Comparison of the absolute difference between ACC and BACC for each model

In this section, we thoroughly explore the impact of imbalanced data on the performance of skin lesion classification models, recognizing the significant challenge it poses for multi-class classification. Imbalanced data often lead to an imbalance in accuracy, with the majority classes dominating and the performance of minority classes being ignored. However, in practical scenarios, the importance of classes may only sometimes align with their prevalence. Particularly in disease identification, detecting positive cases from the minority class holds crucial significance.

To assess the effect of imbalanced data, we examine the absolute difference between accuracy (ACC) and BACC (balanced accuracy) illustrated in Fig. 5. BACC is also the sensitivity of multi-class classification results as shown in Tables 3 and 4. Notably, the absolute difference in the PAD-UFES-20 dataset is smaller than that in the ISIC 2019 dataset. Furthermore, we observe no discernible trend in the absolute difference as the backbone model scale increases.

Moreover, the results demonstrate that LightDPH consistently exhibits a stable difference between ACC and BACC across the two datasets. HAC-LF achieves a similar level of stability to LightDPH; however, it comes at the expense of a larger model scale, approximately twice that of LightDPH. Consequently, the explicit imbalance issues in the large-scale skin lesion dataset contribute to a more substantial disparity between ACC and BACC. Hence, in our evaluation, we prioritize BACC as the primary metric for further analysis of model efficiency, ensuring a balanced consideration of both majority and minority classes.

### Efficiency Analysis of Model Scale and Classification Performance

**Fig. 6:**
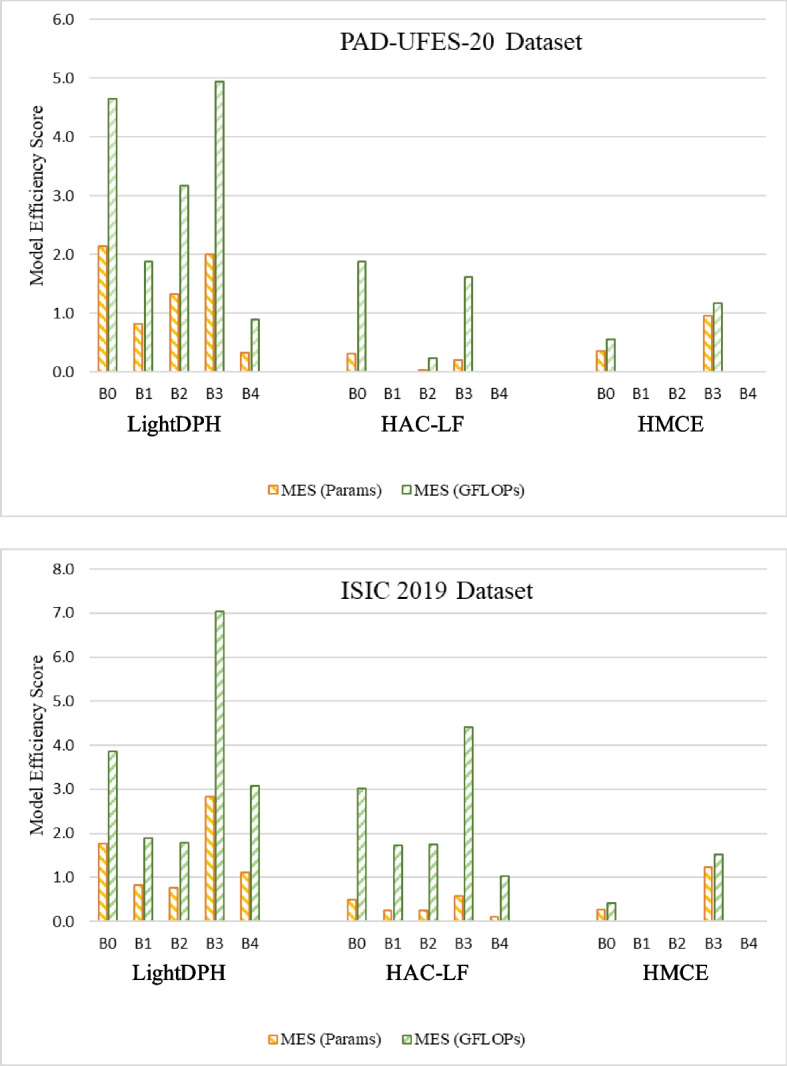
Evaluation of the models with model efficiency scores on PAD-UFES-20 and ISIC 2019 datasets

The evaluation of model scale concerning performance becomes crucial, particularly in medical applications where AI-aided tools serve as physician assistants. Although scaling up the model usually results in better performance for big datasets, it is crucial to evaluate whether the performance gain is worth the effort. In medical applications, achieving comparable performance with smaller model scales holds great promise, especially for portable device deployment. In order to assess the efficiency of inference models, we introduce the concept of model efficiency score (MES). MES allows us to measure the utility of selecting an appropriate model by examining the balance between the number of parameters and floating-point operations and their impact on performance. By considering the harmony between these factors, we can determine the efficiency of a model, facilitating informed decision-making in model selection. The MES provides valuable insights into achieving optimal performance while minimizing the computational burden.

Figure 6 illustrates each model’s MES on the PAD-UFES-20 and ISIC 2019 datasets. MES, calculated according to (8), serves as a metric to assess the efficiency of new models in utilizing increased model scale for performance improvement. In this analysis, BACC is employed as the performance metric, while the number of parameters and GFLOPs serve as variables representing the model scale. BACC is a critical metric for this task as it provides a fair assessment of multi-class classification performance while addressing the challenges of imbalanced data. Furthermore, BACC offers potential for further improvement. The baseline models, EfficientNet-B0 to B4, are the backbone networks for comparison with LightDPH, HAC-LF, and HMCE models.

By examining MES, which reflects the contribution of increased model scale to classification performance, we observe that LightDPH significantly outperforms state-of-the-art methods in terms of efficiency. Notably, there is a substantial disparity in MES (GFLOPs) between LightDPH and the other models. Some MES (Params) and MES (GFLOPs) values are zero, indicating that the performance of those models is either worse or equal to the baseline EfficientNets.

Turning to the MES comparison of the proposed LightDPH and state-of-the-art models on the ISIC 2019 dataset, while HMCE achieves commendable results in major-type classification, it demonstrates limited efficiency due to class imbalance issues, affecting its performance on both datasets. On the other hand, HAC-LF exhibits impressive capabilities in handling large datasets with its extensive floating-point operations. However, the increased number of parameters does not translate to a substantial performance improvement compared to LightDPH. Notably, when comparing MES (Params) and MES (GFLOPs), we consistently find that MES (GFLOPs) exceeds MES (Params) in the evaluation of all models. This observation arises from the discrepancy in floating-point operations being greater than in parameters between the baseline and compared models.

The overall evaluation of performance and model efficiency scores provides valuable insights for practical applications. The results offer constructive guidance for further development and highlight the robustness of LightDPH across different backbone networks on both skin lesion datasets.

## Discussion

This study addresses the requirement for accurate and practical skin lesion classification systems that can be deployed in real-world scenarios. Previous methods relied on ensemble techniques, leading to high storage requirements. In order to address this issue, the study introduces a novel contrastive learning approach, LightDPH, maintaining high accuracy while reducing storage demands. Additionally, the study incorporates hierarchical knowledge to reduce major-type misclassification in skin lesion classification. Compared to the state-of-the-art methods, our proposed LightDPH offers a comprehensive and promising solution for accurate and efficient skin lesion analysis in clinical applications.

In comparison to other state-of-the-art models in terms of model efficiency scores, LightDPH demonstrates the highest model efficiency as the model scale increases based on the baseline model. This finding underlines the exceptional performance of LightDPH in effectively balancing model complexity and classification accuracy. The results conclusively show that as the model scale expands, LightDPH efficiently utilizes its resources, resulting in optimal efficiency. By using various backbone networks, our proposed method exhibits the robustness of ACC and BACC.

Despite the significant contributions and advancements made in this study, there are a few things that could be improved in the context of medical AI research. As highlighted by [37], there is a noticeable gap between the performance of AI methods and their actual implementation in clinical practice, mainly due to the influence of dataset variations. While the findings of this study hold promise for clinical applications, further research and access to comprehensive datasets are essential for bridging this gap and facilitating the effective translation of these insights into clinical practice. Additionally, from a technical perspective, it is worth exploring the performance of self-supervised learning methods that alleviate the reliance on an extensive set of negative samples for training. Previous studies have proposed alternative approaches that effectively address the “collapsing output” issue in self-supervised learning [38, 39]. However, these methods have yet to be widely used in various domains to verify their application competition; thereby, there is a need to advance the field of self-supervised learning and expand its potential impact.

## Conclusion

In this study, we have proposed a novel lightweight hierarchical contrastive learning method with dual projection heads and presented a comprehensive investigation into skin lesion classification using deep learning techniques. Our research focused on developing a lightweight inference model while achieving high model efficiency, which was evaluated by the model efficiency score we proposed. Through extensive experiments and analysis, we have demonstrated the superior performance of LightDPH, which can save more than 50% parameters and 30% GLOPs compared to the state-of-the-art method. We have significantly reduced the model scale required for inference while maintaining the same classification accuracy as HAC-LF. To ensure the robustness and generalizability of our approach. The results consistently demonstrate the effectiveness and superiority of our proposed method across different-scale datasets. This advancement opens up possibilities for deploying skin lesion classification models on resource-constrained devices, such as portable devices and smartphones.

Our initial validation has concentrated on dermatological datasets, emphasizing the effectiveness of our approach in this area. However, the methodology, centered on hierarchical classification and lightweight network architectures, holds significant potential for broader applications. This adaptable approach could be tailored for disease prediction, such as in portable ECG devices, benefiting from similar needs for accurate and efficient classification systems as dermatology. To further enhance its applicability, we plan to deploy our method alongside portable dermatoscopy products and smart devices integrated with dermatoscopes. This strategy will allow us to collect valuable real-world data through field validations involving both medical professionals and general consumers. The insights gained from these validations will be instrumental in refining our model to better meet diverse user needs, ultimately enhancing the user experience across various settings.

As the field continues to evolve, future studies should focus on refining existing methodologies, exploring novel approaches, and addressing any remaining challenges. For instance, the early detection of skin lesions is crucial for prompt treatment [40]. However, the research direction in this area is limited by the scarcity of publicly accessible datasets that provide comprehensive information on the progression of patients’ diseases. From the perspective of AI development in recent years, transformer-based methods have emerged as powerful models for a wide range of domains, including large language models and computer vision [41]. These models have demonstrated exceptional performance on various applications, showcasing their effectiveness in capturing complex patterns and dependencies. However, one limitation of transformer-based methods is their high computational cost compared to conventional CNN-based approaches. Despite this challenge, there is a promising prospect for enhancing large-scale models to enable their practical application in portable and resource-constrained environments in the future.

## Data Availability

All datasets used in this study are available from the following links: PAD-UFES-20 (https://www.kaggle.com/datasets/mahdavi1202/skin-cancer) and ISIC 2019 (https://challenge.isic-archive.com/landing/2019/).

## Code Availability

LightDPH (https://github.com/BXSHIN/lightdph)
